# A pulmonary enteric adenocarcinoma patient harboring a rare EGFR exon 19 P753S mutation: Case report and review

**DOI:** 10.3389/fonc.2022.988625

**Published:** 2022-09-23

**Authors:** Xiaohu Xu, Dong Chen, Xiao Wu, Qi Wang

**Affiliations:** ^1^ Department of Integrated Traditional Chinese and Western Medicine, Tongji Hospital, Tongji Medical College, Huazhong University of Science and Technology, Wuhan, China; ^2^ Department of Pathology, Tongji Hospital, Tongji Medical College, Huazhong University of Science and Technology, Wuhan, China

**Keywords:** pulmonary enteric adenocarcinoma, EGFR mutation, case report, KRAS, TP53

## Abstract

Pulmonary enteric adenocarcinoma (PEAC) is a rare subtype of non–small cell lung cancer (NSCLC), accounting for about 0.6% of all primary lung adenocarcinoma. Although epidermal growth factor receptor (EGFR) mutation is common in primary lung adenocarcinoma, it is rarely reported in PEAC. This case report describes a PEAC patient with co-mutations of EGFR, Kirsten rat sarcoma viral oncogene (KRAS), and TP53, being treated with immunotherapy combined with chemotherapy. A 69-year-old man complained of cough and expectoration with bloody sputum for 2 weeks. The lung-enhanced CT scan showed a massive soft tissue shadow, about 46 × 35 mm in the lower lobe of the right lung. The neoplasm sample in the lower lobe of the right lung was obtained using CT-guided fine-needle aspiration (FNA). Immunohistochemical assays showed that the tumor was positive for CK7, CDX-2, C-MET, and villin. Gastroscopy and rectal colonoscopy had been performed respectively to exclude a diagnosis of colorectal adenocarcinoma. The patient was finally diagnosed with pulmonary intestinal adenocarcinoma. Next-generation sequencing (NGS) analysis showed a rare EGFR exon 19 missense mutation (c.2257C>T, p.P753S), KRAS exon 2 missense mutation (c.35G>T, p.G12V), and TP53 exon 5 missense mutation (c.401T>C, p.F134S). The lung-enhanced CT scan showed that the tumor shrank after four cycles of chemotherapy combined with immunotherapy. We hope that this case report can increase the understanding of this rare type of tumor and provide new molecular indications for diagnosis and individualized treatment. Furthermore, the combination of chemotherapy and immunotherapy seems to be an effective therapy for PEAC. Whether the use of immunotherapy can provide clinical benefits needs to be further explored with more samples in future studies.

## Introduction

Pulmonary enteric adenocarcinoma (PEAC) is a rare subtype of non–small cell lung cancer (NSCLC), which is defined as a morphological enteric cell differentiation involving more than 50% of tumor cells, without evidence of a primary digestive tract tumor (1). It is pointed out that PEAC accounts for 0.6% of all primary lung adenocarcinoma (2). The first case was reported as ‘intestinal type of lung adenocarcinoma’ by Tsao and Fraser in 1991 (3). PEAC was officially listed as a variant subtype of pulmonary invasive adenocarcinoma by the World Health Organization (WHO) in 2015 (4). According to a review, there are no more than 300 cases have been reported as case reports or small case studies in the literature (5). To date, differences between PEAC and conventional lung adenocarcinoma in pathogenesis, lung location type, clinical course, and radiographic features cannot be fully characterized. However, immunohistochemistry (IHC) is still a powerful tool for the diagnosis of PEAC. The rarity of PEAC makes it impossible to provide more information to evaluate the efficacy of different therapeutic methods and prognoses. With the application of next-generation sequencing (NGS) analysis technology in recent years, new perspectives have emerged in delineating new molecular profiles and therapeutic sensitivity of PEAC (6).

The present study reports a case of PEAC with a rare epidermal growth factor receptor (EGFR) exon 19 missense mutation (c.2257C>T, p.P753S), who was initially treated with chemotherapy plus an immune checkpoint inhibitor with satisfactory efficacy. In addition, it reviews PEAC pathological features, gene mutations, and treatments according to the published literature to improve the understanding of this disease. This present study was approved by the Clinical Trial Ethics Committee of Tongji Hospital, Tongji Medical College, Huazhong University of Science and Technology, Wuhan, China. Informed consent was obtained from the patient.

## Case report

**Figure 1:**
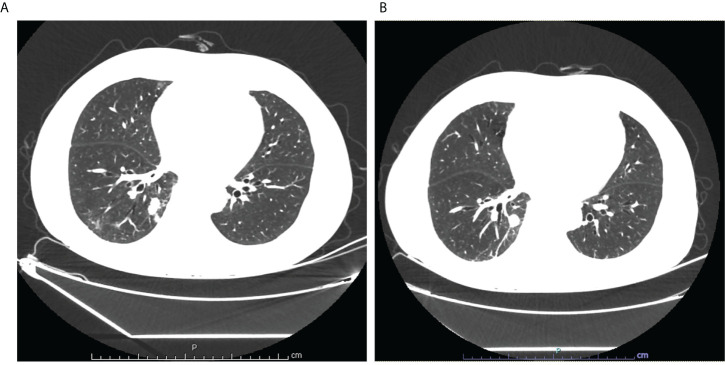
**(A)** Lung enhanced CT scan showed a massive soft tissue shadow, about 46×35mm in the lower lobe of the right lung on April 13; **(B)** Lung enhanced CT scan showed a massive soft tissue shadow, about 44×30mm in the lower lobe of the right lung on July 4.

A 69-year-old non-smoker man was admitted to Tongji Hospital, Tongji Medical College, Huazhong University of Science and Technology (Wuhan, China) on 11 April 2022 and complained of cough and expectoration with bloody sputum for 2 weeks. In March 2022, he visited a local hospital due to cough and expectoration with bloody sputum, but there were no chills, fever, fatigue, or gastrointestinal complaints. A chest computed tomography (CT) scan in the local hospital on 1 April showed a patchy shadow in the right lower lobe. Physicians at the local hospital recommended that the patient should be transferred to the higher-level hospital for further diagnosis and treatment. The patient had a history of kidney calculi disease and denied the history of malignant tumor, other diseases, and surgery. Physical examination at admission showed no obvious positive signs. On 13 April, the lung-enhanced CT scan in Tongji Hospital showed a massive soft tissue shadow, about 46 × 35 mm in the lower lobe of the right lung (**Figure 1A**). To further confirm the diagnosis, the neoplasm samples in the lower lobe of the right lung were obtained using CT-guided fine-needle aspiration (FNA).

**Figure 2:**
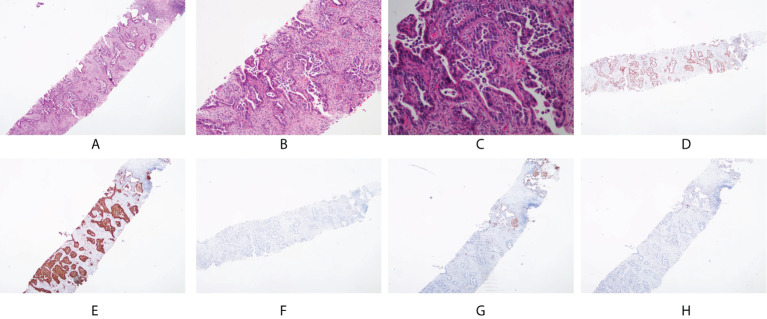
Pathological and immunohistochemical examination results. **(A)** hematoxylin-eosin staining (HE), magnification×40; **(B)** HE, magnification×100; **(C)** HE, magnification×200; **(D)** CDX-2 positivity, magnification×40; **(E)** CK7 positivity, magnification×40; **(F)** CK20 negativity, magnification×40; **(G)** NapsinA negativity, magnification×40; **(H)** TTF-1 negativity, magnification×40.

Immunohistochemical assays showed that the tumor was positive for CK7, CDX-2, C-MET, and villin; focally positive for P40, CD10, MUC6, ROS1, and mesothelin; and negative for thyroid transcription factor-1 (TTF-1), ALK, SATB2(-), MUC2(-), MUC5AC(-), PAX8(-), GATA3(-), PSA(-), NKX3.1(-), napsin A, and CK20 (**Figure 2**). Further pathological examination resulted in a diagnosis of intestinal-type adenocarcinoma of the right lung.

The laboratory data showed that cytokeratin-19 fragment antigen21-1 (CYFRA21-1, 9.57 μg/L) and carbohydrate antigen 19-9 (CA19-9, 106.60 U/ml) levels were increased compared with normal levels. To exclude a diagnosis of colorectal adenocarcinoma, gastroscopy and rectal colonoscopy had been performed respectively. Gastroscopy showed chronic hemorrhagic gastritis and gastric body polyp. Histological examination of the gastric body polyp showed gastric fundus gland polyp. Rectal colonoscopy showed multiple polyps of the sigmoid colon, which was determined as tubular adenoma by histological examination. These pathological and immunohistochemical findings excluded lung metastases of intestinal adenocarcinoma, and the patient was finally diagnosed with pulmonary intestinal adenocarcinoma.

In order to investigate the mutation profile of the neoplasm samples, we obtained the permission of patients to use NGS for gene analysis, which is designed to identify somatic variations of 32 cancer-related genes, including EGFR, KRAS, NRAS, BRAF, RET, PIK3CA, and TP53. Gene analysis of the neoplasm samples was undertaken by GREENIKON Medical Laboratory Co., Ltd (Shanghai, China) using Illumina NovaSeq 6000 platform (Illumina, San Diego, CA). Among the 32 genes examined by this assay, only the EGFR, KRAS, and TP53 genes were mutated. EGFR showed a missense mutation (c.2257C>T, p.P753S) of exon 19. KRAS showed a missense mutation (c.35G>T, p.G12V) of exon 2. TP53 showed a missense mutation (c.401T>C, p.F134S) of exon 5 (**Table 1**). In addition, the expression level of PD-L1 in tumor tissues of patients was detected, and IHC staining results of tissue sections showed that the tumor proportion score (TPS) of PD-L1 was 0%, indicating negative expression. From 30 April, the patient received four cycles of chemotherapy (pemetrexed + cisplatin) combined with immunotherapy (penpulimab). On 4 July, the lung-enhanced CT scan showed that the tumor was smaller than that on 13 April (**Figure 1**).

**Table 1:** Gene mutation result.

Gene	Exon	Nucleotide variation	Amino acid variation	Mutation abundance
TP53	exon5	c.401T > C	p.F134S	19.72%
KRAS	exon2	c.35G > T	p.G12V	15.25%
EGFR	exon19	c.2257C > T	p.P753S	42.80%

## Discussion

As a rare subtype of NSCLC, PEAC has similar clinical manifestations to typical lung cancer, including dry cough, fever, chest/back pain, and hemoptysis. Solid lung masses can be found by CT or PET/CT imaging, and some may be accompanied by speculation, pleural effusion, or indentation radiological findings.

Smoking may be a potential risk factor for lung-intestinal adenocarcinoma, which is reported that 76.9% of PEAC patients had a history of smoking (7). However, another study showed that 46.1% of patients had a history of smoking, and it seemed that there was no significant correlation between PECA and smoking (8, 9). Moreover, a study indicated that PEAC affected more men, but other studies concluded a similar or opposite incidence between men and women (8–10). These controversies may be explained by the limited number of cases reported currently. Nevertheless, there is a general consensus that most PEAC patients are elderly (7–10).

Circulating tumor markers are highly sensitive to distinguish different tumors, but it seems that it is difficult to distinguish PEAC from other tumors. A study found that 68.2% (45/66) patients had elevated carcinoembryonic antigen (CEA) levels, whereas the positive rates of CA125 and CA19-9 were relatively low, with 50% (5/10) and 48.4% (15/31) respectively (9). Another article has suggested that PEAC is associated with increases in serum CEA and CA19-9 (10). Therefore, it is necessary to exclude digestive system tumors by CT, especially gastrointestinal endoscopy.

Although there is no consensus on the characteristics of PEAC, IHC is a great significant tool for the diagnosis. PEAC has similar pathological morphology and IHC features to those of metastatic colorectal adenocarcinoma (MCRC). That is, it has at least one positive enteric differentiation marker including colorectal cancer markers such as caudal-type homeobox 2 (CDX-2), cytokeratin-20 (CK 20), mucin 2 (MUC-2), and villin, whereas lung cancer markers including CK7, napsin A, and TTF-1 can be expressed simultaneously. One study retrieved 33 articles with a total of 170 PEAC patients included in PubMed from 31 January 1991 to 1 August 2020 and found that the positive rate of CK7, CDX2, CK20, and TTF1 was 88.2% (149/169), 78.1% (132/169), 48.2% (82/170), and 38.8% (66/170), respectively (11). Another study reported that the positive expression rates of CK7 and CDX2 were 100%, whereas the positive rates of TTF1, CK20, and MUC2 were 45.6%, 32.6%, and 32.6%, respectively (12). Therefore, CK7 combined with CDX2 was considered to be an important marker for the diagnosis of PEAC.

The present patient was a 69-year-old non-smoker man who complained of cough and expectoration with bloody sputum for 2 weeks. He found a massive soft tissue shadow in the lower lobe of the right lung by a lung-enhanced CT scan. The pathological examination of the tumor tissue obtained by CT-guided FNA revealed a diagnosis of intestinal-type adenocarcinoma of the right lung. Gastrointestinal endoscopy showed that the present patient had multiple polyps, but the pathological examination results ruled out gastric cancer or colon cancer. In the present case study, IHC assays showed that the tumor was positive for colorectal cancer markers (CDX-2, villin) and lung cancer markers (CK7), but negative for TTF-1, CK20, and MUC-2. We also detected the negative expression of SATB 2, because some studies reported that SATB 2 can be used as a negative marker of lung-intestinal adenocarcinoma, so as to differentiate it from lung metastasis of colorectal cancer (13). Based on the above pathological and immunohistochemical results, the present case was diagnosed as PEAC after the primary gastrointestinal tumor was excluded.

Further genetic analysis of PEAC is helpful to provide more information for diagnosis and treatment. At present, most studies believe that the predominant mutations associated with PEAC include KRAS (G12V, G12D, G12C, and G13D) and ALK, whereas EGFR was mostly wild type (8, 10, 12–15). Nottegar et al. found that KRAS was the most common mutation in PEAC, and the mutation rates were 60.9% and 50.0% after analyzing 46 and 8 cases of PEAC, respectively (12). However, KRAS G12V is also common in colonic adenocarcinoma, so KRAS mutation cannot be used to distinguish PEAC from MCRC (13). In these cases, KRAS 12 codon is the most common mutation site, and only one case has KRAS exon 2 mutation and EGFR exon 19 deletion mutation (p.E746_S752). This is also the first report of EGFR mutation in PEAC (12). Since then, many studies have shown that the positive rate of EGFR mutations is about 16.7%, much lower than that of KRAS, and these EGFR mutations are all deletion mutations (8, 10, 12, 13, 16).

Different from what was reported by Nottegar et al., EGFR showed a missense mutation (c.2257C>T, p.P753S) of exon 19 in the present case study. This mutation will result in a change from C to T at base 2,257 of exon 19 of the EGFR gene, resulting in a change from proline (P) to serine (S) at amino acid 753 of the translated protein, which was only reported in a patient with cutaneous squamous cell carcinoma (17). It is speculated that this mutation may lead to exon jumping or protein truncation, thus activating the EGFR kinase domain and increasing the sensitivity to monoclonal antibody inhibition (17). As far as we know, this is the first case report for PEAC with EGFR-p.P753S mutation, although the role of P753 mutation in lung cancer is unclear.

EGFR mutation mainly occurs between exon 18 and exon 21 in NSCLC. The most common mutations include exon 19 deletion and L858R exon 21, which have a convincing response to EGFR tyrosine kinase inhibitors (TKIs). It is reported that 10% of patients with EGFR mutation in NSCLC have uncommon mutations, which include exon 18 mutations, exon 20 insertion mutations, and other rare variants (18). Such as exon 20 insertions and duplications are generally resistant to targeted therapy with TKIs due to the inaccessibility of the binding site for this mutation (19). Although new molecules recently have been approved as subsequent targeted therapies, chemotherapy remains the first-line regimen. In another case, p.E746-A750del and p.delL747-P753insS are the common exon 19 deletion subtypes, which could disrupt inactive conformation of EGFR kinase domain and enhance the effectiveness of EGFR TKIs (20). However, it is not enough to evaluate and determine the survival difference of the exon 19 deletion type as the number of TKI-treated patients was very few. Overall, uncommon mutations are insensitive or have a low response to EGFR-TKIs.

Additionally, we also found KRAS missense mutation (c.35G>T, p.G12V) of exon 2 in this case. KRAS gene is a proto-oncogene in the RAS family, and its most frequent mutation sites are almost all concentrated in codon 12 of exon 2, accounting for about 90% (21). The KRAS gene is a downstream factor of the EGFR signaling pathway. The continuous activation mutation of KRAS may affect the therapeutic effect of EGFR-TKIs in NSCLC patients. Pan analyzed the results of 41 global studies and found that the overall prognosis of NSCLC patients would be worse when KRAS mutation was found, and KRAS mutation was closely related to EGFR-TKIs resistance (22). In addition, TP53 showed a missense mutation (c.401T>C, p.F134S) of exon 5. TP53 is an important tumor suppressor gene in cells. It has the highest mutation frequency (>50%) in many different types of tumors. It is a negative regulator in the cell growth cycle, which is related to important biological functions such as cell cycle regulation, cell apoptosis, cell differentiation, DNA repair, and angiogenesis (23, 24). Most of the TP53 mutations are missense mutations, accounting for more than 75% of the total mutations, and exons 5–8 are the most common mutation sites (25, 26). As the most common type of co-mutation in advanced NSCLC, studies have shown that EGFR-TP53 co-mutation may weaken the therapeutic effect of TKIs (27). Because mutations of EGFR are rare in PEAC, there have been no reported cases of TKIs. Therefore, the co-mutations of EGFR, KRAS, and TP53 present in this case suggest that this patient is not suitable for targeted drug therapy.

For the treatment of this PEAC patient, we chose PP chemotherapy because it was reported that one stage IV PEAC patient was treated with four times pemetrexed + cisplatin, and tumor evaluation was PR (28). Another patient was treated with six times pemetrexed + carboplatin, and tumor evaluation was SD (29). Based on the above exploration of chemotherapy, this study case was treated with pemetrexed and carboplatin, and the fourth cycle of chemotherapy has been completed. Immunotherapy is a breakthrough in tumor therapy in recent years, and its efficacy has been confirmed in various tumors. Studies have shown that PD-1 is highly expressed in PEAC, and compared with ordinary lung adenocarcinomas, non-synonymous tumor mutational burden (TMB) is significantly higher in patients with PEAC, indicating that patients with PEAC may benefit from immune checkpoint inhibitors (10). Therefore, it is very important to determine whether PEAC patients may benefit from immunotherapy. After informing the benefits and possible side effects of immunotherapy, the patient agreed to PP chemotherapy combined with penpulimab immunotherapy (30). The lung-enhanced CT scan showed that the tumor shrank after four cycles of chemotherapy combined with immunotherapy. At the time of drafting the present article, the patient is still alive and about to undergo the next cycle of treatment.

In conclusion, the present study reports the first case of PEAC with a rare EGFR exon 19 missense mutation (c.2257C>T, p.P753S), KRAS exon 2 missense mutation (c.35G>T, p.G12V), and TP53 exon 5 missense mutation (c.401T>C, p.F134S). Immunohistochemical targets and gene mutations observed in this patient may be helpful in the diagnosis, treatment, and prognosis of patients with PEAC. Furthermore, the combination of chemotherapy and immunotherapy seems to be an effective therapy for PEAC. Whether the use of immunotherapy can provide clinical benefits needs to be further explored with more samples in future studies.

## Data availability statement

The datasets for this article are not publicly available due to concerns regarding participant/patient anonymity. Requests to access the datasets should be directed to the corresponding author.

## Ethics statement

The studies involving human participants were reviewed and approved by the Clinical Trial Ethics Committee of Tongji Hospital, Tongji Medical College, Huazhong University of Science and Technology, Wuhan, China. The patients/participants provided their written informed consent to participate in this study. Written informed consent was obtained from the individual(s) for the publication of any potentially identifiable images or data included in this article.

## Author contributions

QW designed the idea. DC performed the pathological imaging. XXH and XW collected the data and wrote the manuscript. All authors read and approved to submit the report for publication.

## Funding

This work was supported by the 6th Young Talent Lifting Project of China Association For Science and Technology (No.YESS20200255).

## Acknowledgments

The authors would like to thank the patient and obtain the written informed consent provided by the patient for publishing the report.

## Conflict of interest

The authors declare that the research was conducted in the absence of any commercial or financial relationships that could be construed as a potential conflict of interest.

## Publisher’s note

All claims expressed in this article are solely those of the authors and do not necessarily represent those of their affiliated organizations, or those of the publisher, the editors and the reviewers. Any product that may be evaluated in this article, or claim that may be made by its manufacturer, is not guaranteed or endorsed by the publisher.

## Glossary

**Table:**

PEAC	Pulmonary enteric adenocarcinoma
NSCLC	Non–small cell lung cancer
EGFR	Epidermal growth factor receptor
KRAS	Kirsten rat sarcoma viral oncogene
TP53	Tumor protein p53
CT	Computer Tomography
FNA	Fine-needle aspiration
CK7	Cytokeratin-7
CDX2	Caudal-type homeobox 2
C-MET	Mesenchymal-epithelial transition factor
NGS	Next-generation sequencing
WHO	World Health Organization
CD10	Lymphocyte antigen 10
ROS1	Receptor tyrosine kinase
TTF-1	Thyroid transcription factor-1
ALK	Anaplastic lymphoma kinase
SATB2	Special AT-rich sequence binding protein 2
MUC2	Mucin 2
MUC6	Mucin 6
MUC5AC	Mucin 5AC
PAX8	Paired Box 8
GATA3	GATA binding protein 3
PSA	Prostate-specific antigen
NKX3.1	NK3 homeobox 1
CK20	Cytokeratin-20
CYFRA21-1	Cytokeratin-19 fragment antigen21-1
CA19-9	Carbohydrate antigen 19-9
ECT	Emission Computed Tomography
TPS	Tumor proportion score
PD-L1	Programmed death ligand 1
CA12-5	Carbohydrate antigen 12-5
TKIs	Tyrosine kinase inhibitors
PR	Partial response
SD	Stable disease
PFS	Progression-free survival
OS	Overall survival
